# Emergence of dynamic properties in network hypermotifs

**DOI:** 10.1073/pnas.2204967119

**Published:** 2022-08-01

**Authors:** Miri Adler, Ruslan Medzhitov

**Affiliations:** ^a^Broad Institute of MIT and Harvard, Cambridge, MA 02142;; ^b^HHMI, Yale University School of Medicine, New Haven, CT 06510;; ^c^Department of Immunobiology, Yale University School of Medicine, New Haven, CT 06510;; ^d^The Tananbaum Center for Theoretical and Analytical Human Biology, Yale University School of Medicine, New Haven, CT 06510

**Keywords:** emergence, feedforward loops, feedback, mathematical modeling, systems biology

## Abstract

Networks are fundamental for our understanding of complex systems. The study of networks has uncovered common principles that underlie the behavior of vastly different fields of study, including physics, biology, sociology, and engineering. One of these common principles is the existence of network motifs—small recurrent patterns that can provide certain features that are important for the specific network. However, it remains unclear how network motifs are joined in real networks to make larger circuits and what properties emerge from interactions between network motifs. Here, we develop a framework to explore the mesoscale-level behavior of complex networks. Considering network motifs as hypernodes, we define the rules for their interaction at the network’s next level of organization. We develop a method to infer the favorable arrangements of interactions between network motifs into hypermotifs from real evolved and designed network data. We mathematically explore the emergent properties of these higher-order circuits and their relations to the properties of the individual minimal circuit components they combine. We apply this framework to biological, neuronal, social, linguistic, and electronic networks and find that network motifs are not randomly distributed in real networks but are combined in a way that both maintains autonomy and generates emergent properties. This framework provides a basis for exploring the mesoscale structure and behavior of complex systems where it can be used to reveal intermediate patterns in complex networks and to identify specific nodes and links in the network that are the key drivers of the network’s emergent properties.

## Significance

Complex systems with multiple components that influence each other, from societies to interacting genes, can be studied using a network description. The nodes in a network are the atoms. Just as atoms combine into molecules that have new properties, nodes combine into network motifs—building-block patterns that frequently recur in the network and have certain dynamic properties. But how are these building blocks combined in networks and what properties emerge when they interact with each other? Here, we define hypermotifs—arrangements of network motif assemblies. We identify enriched hypermotifs in real networks and find that each type of network is enriched in specific hypermotifs that show new dynamic properties. This framework defines the next level of organization in complex networks.

**Fig. 1.**
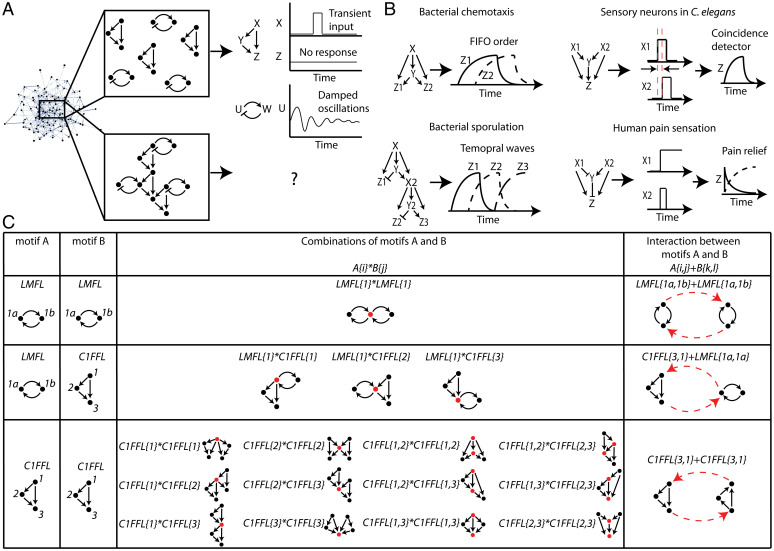
A framework to explore hypermotifs in complex networks. (*A*) Network motifs such as the coherent type 1 FFL (C1FFL) and the mutual feedback loop where u is an activator and w is a repressor can filter out transient input signals and generate damped oscillations, respectively. However, it is unclear what would be the properties of the circuits made up of combinations of different network motifs. (*B*) Examples of four real networks in which the FFL is a network motif, but the way it is joined with other FFLs is different and provides distinct dynamical properties. (*C*) A table that exemplifies the two ways in which network motifs can be directly joined together for the Lock-ON mutual feedback loop (LMFL) and the coherent type 1 FFL (C1FFL). Combination of motifs A and B is where the two motifs share at least one node. These are marked by A{i}*B{j}, where {i}, {j} is the set of nodes each motif is sharing. Interaction of motifs A and B is where the two motifs are linked through at least one edge, A{i,j}+B{k,l}, where i and k are the set of sender nodes, and j and l are the set of receiver nodes from motifs A and B, respectively. The shared nodes in the “Combinations” column and the linking edges in the “Interactions” column are marked in red.

Complex systems describe a collection of multiple agents that influence each other. A common and powerful tool in exploring the structure of complex systems is the use of a network description, where the nodes represent the individual agents and the edges represent the interactions between them (1–6). Interactions between individual nodes in networks generate network motifs—small patterns that are significantly enriched in real networks compared to randomized networks (7). Network motifs can be considered the network’s building-block components, providing certain dynamical properties. Network motifs such as the feedforward loop (FFL) have been studied separately both theoretically and experimentally in various fields and their dynamical properties were elucidated (8–10). However, it remains unclear how network motifs are arranged within real networks into larger patterns and what properties can emerge from these higher-level functional modules (Fig. 1*A*).

Indeed, it has been established that although the same network motifs appear in different contexts, the way that they are joined varies to provide distinct features. For example, FFLs are joined in bacterial transcription networks with multiple outputs to produce a first-in-first-out response. During sporulation, *Bacillus subtilis* uses cascades of FFLs to activate genes in a series of temporal waves. In neuronal networks, FFLs can combine with multiple inputs to provide coincidence detection and pain relief when multiple pain sensation inputs are integrated into a single output (11, 12) (Fig. 1*B*). Thus, combining the same kind of canonical motifs in different ways can generate novel properties and can also be used to “silence” certain motif properties when they are not needed.

Work on modularity (13–15) and networks of networks (16) has revealed hierarchical structures (17) where several levels of organization are sometimes needed to describe the network. Recently, Battiston et al. (18) reviewed existing approaches to explore higher-order interactions in complex networks including the use of hypergraphs and simplicial complexes (19) and highlighted the challenges in the field where higher-order interactions are hard to infer from real data that are mostly based on simple pairwise interactions. Previous studies have explored higher-order clusters and generalizations of network motifs (20–23) and studied network motifs at different scales in real networks (24). However, there are currently no approaches to explore how building-block circuits such as network motifs interact with each other in the network based on specific circuit topologies to form the next level of organization. In order to understand the origin of emergent properties in complex systems it is important to study how network motifs interact with each other as defining the rules of atom interactions that form molecules was crucial for the understanding of the macroscale behavior of matter.

Here, we develop a framework for exploring the rules for emergent properties at intermediate levels of organization of complex networks. In this framework, we consider that the individual agents that interact with each other are the minimal building-block circuit topologies in the network and explore the emergent properties that result from the way that they are embedded in the network, which we call hypermotifs. We develop a method to explore the favorable arrangements of these hypermotifs in real networks and apply it to biological, neuronal, social, linguistic, and electronic networks. This approach sheds light on the inner structure of complex networks and reveals levels of organizations in real evolved and designed networks.

## Results

### A Framework for Exploring High-Level Modules of Network Motifs.

To explore how two network motifs are integrated to form hypermotifs—higher-level network modules—we consider two ways in which motifs can be directly joined. The first is where the two motifs share at least one node, which we define as a combination of motifs. The maximal number of shared nodes ($N_{V,\;\max}$) must be smaller than the size of the smallest motif embedded such that the autonomy of each motif’s topology is maintained. Therefore, $N_{V,\;\max}=\min(n_{A},n_{B})-1$ where $n_{A}$ and $n_{B}$ are the sizes of motifs A and B, respectively. The identity of the nodes that are shared between the motifs is based on categorizing the nodes in each motif according to their unique roles. For example, nodes that participate in FFLs will be categorized into three distinct groups: 1) input, 2) intermediate, and 3) output nodes. If the roles in a certain motif are symmetric, we consider them in the same category (21). We mark a combination of motifs A and B in which nodes {*i*} of motif A are shared with nodes {*j*} of motif B as A{*i*}*B{*j*} (Fig. 1*C*).

The second way two network motifs can be joined, which we define as an interaction between motifs, is when they are linked through at least one edge. When two motifs interact, every pair of nodes that do not participate in the same motif can be linked. Therefore, the maximal number of linking edges is $N_{E,\;\max}=2n_{A}n_{B}$ for directed networks, and half as much for undirected networks. We mark an interaction between motifs A and B as A{*i,j*}+B{*k,l*}, where there are links from nodes {*i*} of motif A to nodes {*l*} of motif B and links from nodes {k} of motif B to nodes {j} of motif A (Fig. 1*C*).

In Fig. 1*C* we exemplify these definitions for several pairs of motifs. Two mutual feedback circuits can only be combined by sharing one node. A mutual feedback circuit and an FFL can be combined by sharing one node which can be either the FFL’s input, intermediate, or output node. The space of possible combinations increases substantially as the size of the motifs that are being combined increases. For example, two FFLs have 12 different ways to be joined when they share either one or two nodes. Note that we list in Fig. 1*C* the core topology of each possible combination. However, each such combination can be extended where every pair of nodes that do not participate in the same motif can be linked (*Materials and Methods* and *SI Appendix*, Fig. S1). For each pair of motifs in Fig. 1*C*, we provide an example of the way they can interact (see *Materials and Methods* for all possible topologies of motif interactions and *SI Appendix*, Fig. S1). This framework thus provides a way to count all possible circuit topologies for combinations and interactions of two motifs.

### Observed Combinations of Network Motifs in Real Networks.

**Fig. 2.**
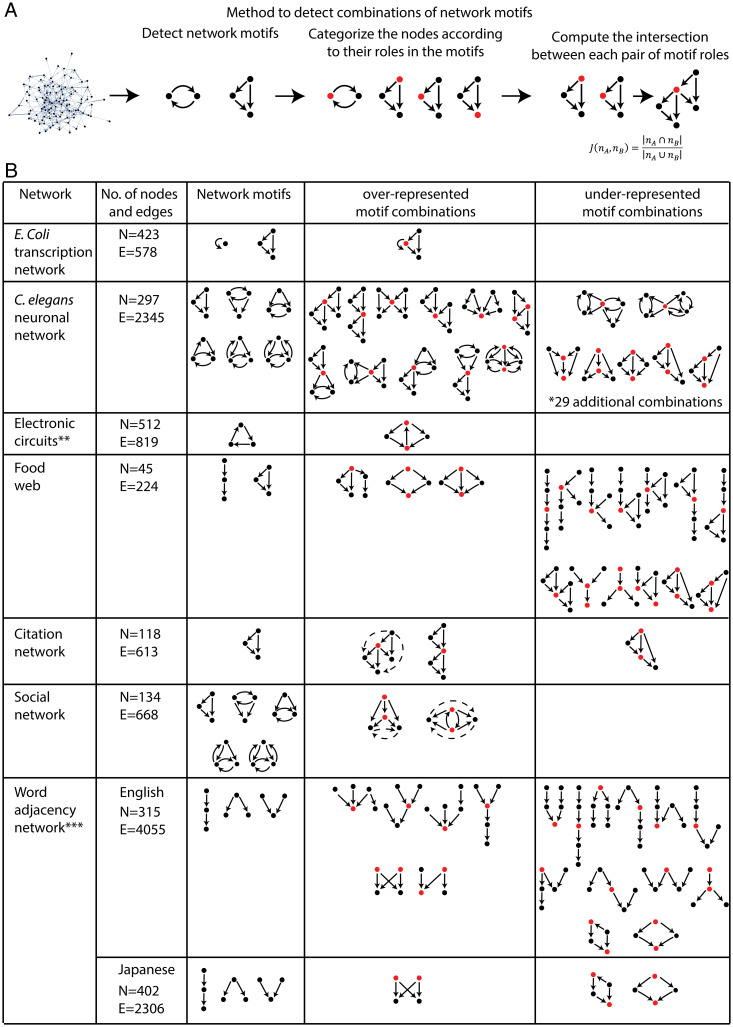
A method to detect enriched combinations of network motifs in real networks. (*A*) Schematics of the method to detect enriched combinations of network motifs in real networks. (*B*) A table that summarizes the analysis of real networks with the type of network, its number of nodes and edges, its network motifs, and over- and underrepresented combinations. Shared nodes are marked in red. We list in *SI Appendix*, Fig. S3*B* the full list of underrepresented combinations of the *C. elegans* neuronal network. In the citation and social networks, the dashed arrows in the overrepresented combinations represent additional edges that are not part of the network motifs in the combined circuit but often appear in the network (*SI Appendix*, Fig. S3*A* for their frequencies in the networks). **We analyzed three different electronic circuits. The combined circuit in which two three-node loop circuits share an edge is significantly enriched in all of them. The other two electronic circuits have *n* = 122, E = 189 and *n* = 252, E = 399. ***We downsampled the word adjacency networks (see *Materials and Methods* for details about the downsampling method we used).

To explore whether network motifs are joined in real networks in specific ways, we developed a method to detect enriched combinations of network motifs in large networks. Given a real network of interest, the first step is to detect the network motifs (up to size n) that characterize the network. There are several algorithms for finding network motifs in large networks (7, 25, 26). After the network motifs of up to n-order have been identified, we categorize the nodes that participate in the network motifs based on their role in the motifs. Next, we compute the level of overlap between nodes in every pair of motif roles. A large overlap between two groups of motif roles means that the motifs are often combined in the network by sharing these nodes (Fig. 2*A*). Finally, in order to test the statistical significance of these combinations, we compare the observed overlap in the real network to the overlap in randomized networks when we keep properties of the network including the degree distribution and the frequency of all subgraphs up to size n the same as in the real network. This comparison allows us to find statistically significant over- and underrepresented combinations of network motifs that do not emerge due to topological constraints in the network (27, 28) (*Materials and Methods*). We highlight that detecting enriched hypermotifs is different from simply detecting larger motifs. Hypermotifs are composed of elementary motifs. Therefore, hypermotifs are a subset of all possible larger motifs. In *SI Appendix* we apply the method to detect hypermotifs on synthetic networks that we generated with specified distributions of network motifs and hypermotifs (*SI Appendix*, Fig. S2).

We applied this method for several natural and designed networks of different origin. In the *Escherichia coli* transcription network (29) the nodes are transcription factors and their target genes, and the edges represent regulatory interactions. In this network the self-loop and FFL motifs are often combined such that the intermediate node of the FFL shows autoregulation. In the neuronal network of *Caenorhabditis elegans* (7, 30) where edges represent synaptic connection between neurons, there are six network motifs including the FFL and five different versions of mutual feedback circuits. We find a large number of overrepresented combinations of these motifs in the network, in line with the abundance of evidence for high-order circuits in neuronal systems (31, 32). The overrepresented combinations show that the neuronal network has a layered structure where in most cases an output node of one motif serves as an input of another motif. Moreover, most combinations in which two motifs are not combined in a layered way are excluded in the network. One exception to this structure is a combination where two double mutual feedback circuits are intertwined. We discuss potential emergent properties of this combined circuit in the next section. Interestingly, there is a large number of combinations that are excluded in the *C. elegans* neuronal network (*SI Appendix*, Fig. S3), supporting the notion that network motifs are not distributed randomly in the network but are arranged in a way that provides a desired functionality in a given system.

In an electronic circuit network (digital fractional multipliers) (33, 34) where the nodes represent different logic gates and flip-flops, the three-node feedback loop circuits are often combined by sharing two nodes with other three-node feedback loop circuits. In a food web of lizards on the St. Martin island (35) where the edges represent predator–prey relations, the network motifs are a three-node chain and an FFL. These motifs are often combined in the network where they either share the highest predator or the lowest prey in the motifs, or both. The network motifs in this food web tend not to share intermediate-level predators or preys and avoid very long food chains.

In a citation network (36, 37) where nodes are researchers and edges represent scientific citations, the FFLs are arranged in cascades. This structure is in line with the fact that citation networks are a type of an information network where the flow in the network is possible only in one direction. The over- and underrepresented combinations in the citation network further show that scientists tend to cite recent papers more than original ones. We considered a social network (38) where nodes are people and an edge is drawn from person A to person B if person A considers person B as a close friend. In this friendship network, the network motifs include the FFL and four versions of mutual feedback circuits. These motifs are often combined such that two FFLs share the input and intermediate nodes, and two regulating mutual feedback loops share the nodes that mutually interact with each other. In these enriched combinations the “output” nodes usually show mutual links in the network. This suggests a pattern where popular people are liked by either members of the same clique or individuals that do not show a mutual friendship. Finally, we analyzed word adjacency networks of texts in English and Japanese where each node represents a word and a directed connection occurs when one word directly follows the other in the text (39). The network motifs in both languages are a three-node cascade, a regulating and regulated V circuits. We find that these motifs show two distinct patterns where they either combine in a layered manner or that certain words have multiple words that are adjacent to them (Fig. 2*B* and *Materials and Methods*). Interestingly, the examination of combinations of network motifs in these linguistic networks shows that there are common principles in the way the network motifs are integrated where the enriched and excluded combinations of network motifs in the Japanese text are also found in the English network. See *SI Appendix*, Fig. S3 for information on the statistical significance of all overrepresented combinations we detected in real networks. Identifying network hypermotifs in real networks thus reveals specific patterns that the network motifs are often combined into and sheds light on the inner structure of the network.

### Emergent Properties of Combinations of Building-Block Circuits.

**Fig. 3.**
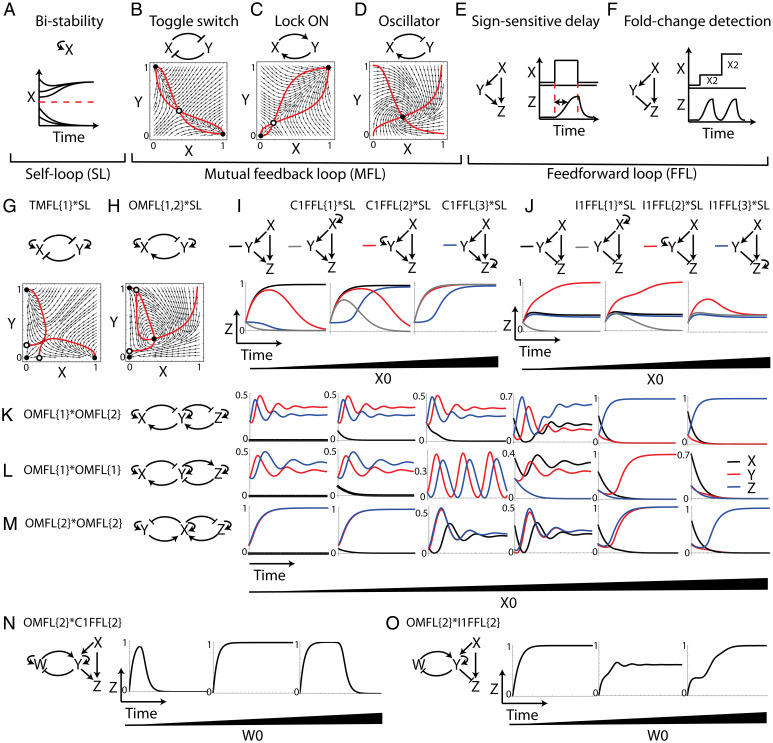
Emergent dynamical properties of combinations of network motifs. (*A–F*) Three classes of circuits that we consider as minimal building blocks and examples of their dynamical properties for given parameter values. In the phase portraits of the mutual feedback circuits the black circles represent stable fixed points and the white circles represent unstable fixed points. The corresponding equations for each panel are described in *Materials and Methods*. (*G* and *H*) Combinations of self-loop and mutual feedback circuits and their phase portraits. (*I* and *J*) Combinations of self-loop and coherent (*I*) or incoherent (*J*) FFLs and their output Z dynamical behavior. We used initial conditions of $X_{0}=0,0.1,0.5,\;Y_{0}=0.185,\;Z_{0}=0.19$. (*K*–*M*) Three different combinations of two oscillator circuits and their dynamical properties when the initial level of X varies. We used $Y_{0},\;Z_{0}=0.2$ throughout and $X_{0}=0,0.1,0.2,0.4,0.6,0.7$ for each combination. (*N* and *O*). Combinations of an oscillator circuit and a coherent (*N*) or an incoherent (*O*) FFL and their output Z dynamical behavior. We used $X_{0},\;Z_{0}=0.01,Y_{0}=0.3$ throughout and $W_{0}=0.1,1,10$ for each combination.

We next explore the dynamical properties of combinations of network motifs. In our modeling framework, we use nonlinear Hill functions to describe relations between the nodes in a circuit (*Materials and Methods*). This modeling approach describes the relationship between nodes in biological networks such as gene regulatory, signaling, and neuronal networks. Other types of networks such as social and linguistic networks require different modeling assumptions. As our minimal building-block circuits, we consider three classes of circuit topologies that were found as network motifs in real natural and engineered networks (11). The first is a self-loop circuit which is a simple motif with only one node (X) positively autoregulating its own levels that can provide bistability. This means that X converges to a high steady-state level only if its initial level is above a certain threshold, and otherwise it declines to zero (Fig. 3*A*). The second class of circuits we consider includes three types of mutual feedback circuits: the toggle switch circuit (TMFL) in which X and Y mutually inhibit each other, leading to a switch between their final levels (Fig. 3*B*), the Lock-ON circuit (LMFL) in which X and Y are both turned ON or OFF due to their mutual activation (Fig. 3*C*), and the oscillator circuit (OMFL) with X as a repressor and Y as an activator (Fig. 3*D*). The third class of circuits is the FFL circuit with two main types: a coherent type 1 FFL (C1FFL) in which the input (X) activates an intermediate node (Y) and both X and Y activate the output (Z) (Fig. 3*E*) and an incoherent type 1 FFL (I1FFL) where Y is a repressor of Z (Fig. 3*F*). We show in Fig. 3 *E* and *F* examples of previously explored dynamical features that the FFL circuits can exhibit for a wide range of parameters (*Materials and Methods*). These dynamical properties were experimentally measured in various contexts ranging from transcription networks in bacterial cells to organismal-level sensory systems (40, 41). We note that in general when we discuss a circuit’s dynamical properties we are referring to properties that are observed for a given choice of models (e.g., nonlinear relations, AND vs. OR logic gates) and a certain range of model parameters. We therefore chose parameters for which the circuit in question shows a typical dynamical behavior (e.g., bistability for the SL, pulsatile behavior for the I1FFL, etc.). However, the circuits may show other properties for other choices of models or parameter values (42, 43). When we discuss the properties of a combination of circuits, we compare them to the properties of each circuit component when we keep the same choice of parameter values.

Combining a self-loop motif with other motifs provides bistability to the node that is positively self-regulating. Therefore, a toggle-switch circuit combined with self-loops gives rise to a new stable state in which both X and Y are turned OFF (Fig. 3*G*). The oscillator circuit with both nodes self-regulating shows two additional stable steady states: an OFF state where both X and Y are turned OFF and a state where only Y (the activator) is turned ON. A state where only X (the repressor) is turned ON is not possible since the repressor cannot increase its levels without the presence of the activator Y (Fig. 3*H*). A Lock-ON circuit combined with self-loops does not provide new steady states since bistability is already provided in a simple Lock-ON circuit. Here we focus on a positive self-loop motif that can provide new steady states. A negative self-loop motif combined with other motifs does not provide bistability but can accelerate the circuits’ response time (44).

When exploring combinations of a self-loop motif and FFL circuits, we asked whether the FFL’s behavior is sensitive to the identity of the node that has a self-loop. To that aim, we compared the dynamics of the output Z of a simple FFL (without self-loops) to FFLs with a self-loop on the FFL’s input, intermediate, or output nodes. The FFL combined with self-loops provides bistability to the FFL’s response where the final level of the output depends on the initial level of the input. However, FFL circuits in which the output node is autoregulated are less sensitive to the initial levels of the input (Fig. 3*I*). The incoherent FFL shows an interesting nontrivial behavior when the self-loop is on the intermediate repressor, Y. For high initial levels of input, the output shows a pulsatile behavior, which is similar to the behavior of a simple incoherent FFL. However, if the initial levels of input are low, the output rises to a high steady-state level with a delayed response. The reason for this behavior is that for low levels of input the repressor Y declines to zero due to its self-loop, thus lifting the repression on the output Z and allowing it to increase its levels (Fig. 3*J*). The emergence of this new high steady-state level is possible only if the intermediate node is the one that is positively autoregulated (*Materials and Methods*). Thus, the self-loop provides an additional thresholding mechanism for the behavior of the dynamical circuits it is coupled with. This mathematical model therefore provides a possible explanation for why the FFL and self-loop motifs are combined in the *E. coli* transcription network such that the FFL’s intermediate node has a self-loop interaction. This combination may provide bistability where the *E. coli* target genes are sensitive to the levels of the input signal.

We next show that different combinations of the same two motifs can yield different dynamical properties when keeping the same parameter values. To demonstrate this, we consider three different combinations of two oscillator circuits. Exploring the dynamical behavior of the combinations of circuits for varying initial conditions shows that the different combinations behave dynamically differently and converge to different steady states (Fig. 3 *K–M* and *Materials and Methods*).

We find that coupling the oscillator circuit with the coherent and incoherent FFLs through their intermediate node yields interesting features of a pulsatile response (for a coherent FFL) or a delayed rising response (for an incoherent FFL) for low and high initial levels of W. The width of the pulse and the duration of the delay are proportional to the initial levels of W (Fig. 3 *N* and *O* and *Materials and Methods*). In *SI Appendix*, Figs. S4 and S5 we show the modeling results of the combinations of all possible pairs of circuits from these three classes of circuit topologies.

There are several observations that this framework demonstrates when one considers the potential emergent properties of combinations of network motifs. First, the way that the motifs are combined or the identity of the shared nodes that link the two motifs is an important factor that can drastically affect the resulting behavior. Second, we find that when multistablity emerges from combining two motifs, oftentimes the combined circuit can both preserve the autonomy of each circuit component where it shows their individual properties and show emergent properties (properties not present in individual motifs), depending on the initial conditions.

### Emergent Properties of Combinations of Network Motifs in Real Networks.

**Fig. 4.**
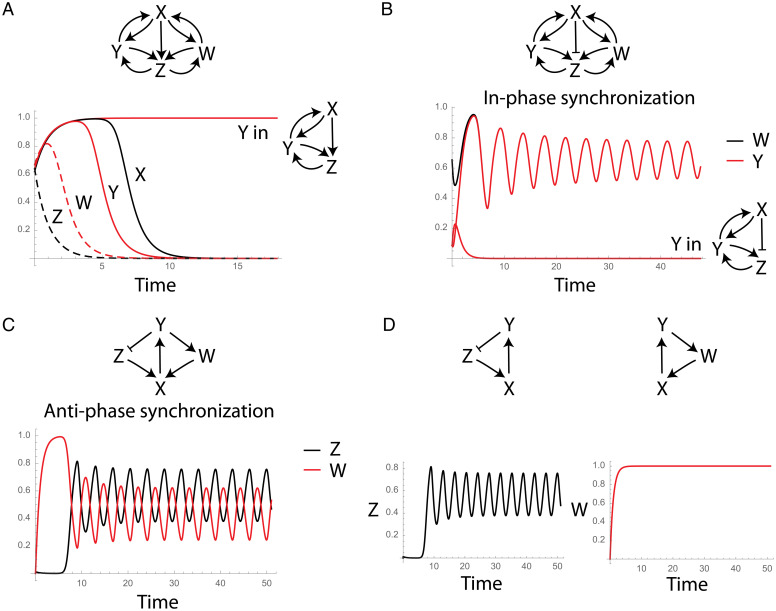
Overrepresented network motif combinations in real networks provide emergent properties. (*A*) Dynamical behavior of the *C. elegans* overrepresented combination of two double mutual feedback circuits where all arrows are positive interactions. Y from the separated X, Y, Z circuit with the same parameters would have converged to a high steady-state level. (*B*) Dynamical behavior of the *C. elegans* overrepresented combination of two double mutual feedback circuits where X inhibits Z. Y from the separated X, Y, Z circuit with the same parameters would have declined to zero without oscillations. (*C*) Dynamical behavior of the electronic circuits over-represented combination of two three-node loop circuits where all edges are positive except for one where Y inhibits Z, where it shows antiphase synchronization. (*D*) W from the separated X, Y, W circuit with the same parameters (or any other choice of parameters; *Materials and Methods*) does not show oscillations.

We next model the dynamical behavior of several overrepresented combinations of network motifs that we detected in real networks in order to exemplify the potential emergent properties that may result from these combinations. The first combination of motifs that we model is the double mutual feedback motif from the *C. elegans* neuronal network (Fig. 2*B*). In this motif there are two pairs of neurons that mutually interact with each other (X, Y and Y, Z) while X interacts with Z only in one direction. We find that this motif is often combined in the neuronal network with other motifs of the same type where the one-direction edge from X to Z is shared between the two motifs. Considering that the interactions in the motif are all positive, each motif separately is a generalization of the Lock-ON feedback circuit where it can provide bistability such that X, Y, and Z are all either turned OFF or ON. Combining two such double mutual feedback motifs link the fates of the Y and W neurons from both motifs and can also provide a temporal order for their pulsatile behavior (Fig. 4*A* and *Materials and Methods*). We also model the same motif combination where we consider that X inhibits the activity of Z. Here, each double mutual feedback can provide oscillations for a certain range of model parameters or converge to an OFF state. When the two motifs are combined such that the X-to-Z inhibitory edge is shared, the oscillations of one circuit propagate to the neuron that participates in the other motif although it would not have shown oscillations autonomically with the same parameter values. Moreover, it will synchronize and have the same phase as the motif it is linked with, demonstrating in-phase synchronization, which is an important property of neuronal networks (45) (Fig. 4*B* and *Materials and Methods*).

The second combination of motifs that we model is of the three-node feedback loop that was found to be enriched in the electronic circuits network (Fig. 2*B*). A combination of two three-node loop circuits where one has three positive interactions (all-positive-interactions circuit) and the other has one negative and two positive interactions show emergent properties (Fig. 4*C*). Here the oscillations of the circuit with the negative interaction propagate to the second all-positive-interactions circuit where it oscillates with an opposite phase showing antiphase synchronization. We note that here the emergence of oscillations in the all-positive-interactions circuit is especially interesting since on its own this circuit does not show pure oscillations even for a different choice of model parameters (Fig. 4 *C* and *D* and *Materials and Methods*).

These examples illustrate the complexity that can emerge from simple combinations of minimal building-block circuits in real networks.

### Emergent Properties of Interactions of Network Motifs.

**Fig. 5.**
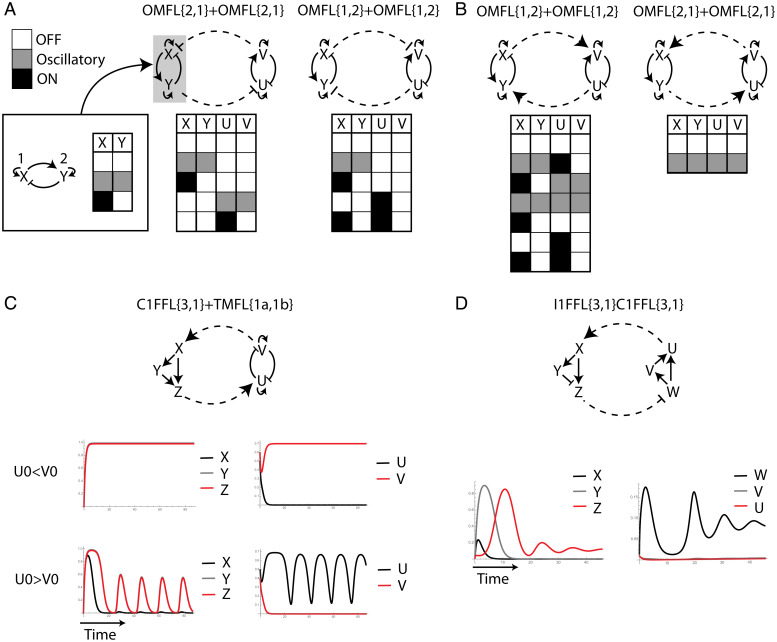
Emergent dynamical properties of interactions of network motifs. (*A* and *B*). Examples of interactions of two oscillator feedback circuits where they mutually repress each other (*A*) and mutually activate each other (*B*). The stable steady states for the same choice of model parameters for all four circuits are shown where white squares represent the OFF state, black squares are shown when a variable is fully turned ON, and the gray squares represent damped oscillations around an intermediate level steady state. (*C*) An example of a mutual activation interaction between a coherent FFL and a toggle-switch feedback circuit with the dynamic behavior of all variables for two different initial conditions with the same model parameters (*Materials and Methods*). (*D*) An example of an interaction between two FFLs where the coherent FFL is an activator and the incoherent FFL is a repressor and the dynamic behavior of all variables. See *Materials and Methods* for the equations we used for all interactions of motifs.

The second way of joining two network motifs that we consider is an interaction between network motifs. Here, every motif can be considered as a hypernode in a higher-level circuit. Interaction of network motifs can thus show properties of circuits at two levels: the high-level circuit topology in which the motifs are single nodes and the properties of the low-level circuits that are being interconnected. To illustrate this, we model an interaction of two oscillator circuits in a toggle-switch high-level topology. We find that this module shows a toggle switch between the properties of the oscillator circuits. However, the identity of the connecting nodes in each circuit and the choice of parameter values may influence the properties of the new high-level module (Fig. 5*A* and *Materials and Methods*). Similarly, two oscillator circuits that mutually activate each other can show all possible combinations of their individual steady states, which is a high-level version of the Lock-ON circuit property where the motifs are either both turned OFF or ON (Fig. 5*B* and *Materials and Methods*).

Interaction between two motifs can sometimes lead to emergent properties that cannot be observed in each of the motifs separately. For example, a coherent FFL and a toggle-switch feedback circuit that mutually activate each other can exhibit oscillations which are not a property of an FFL or a toggle-switch circuit (*Materials and Methods*). Joining them in this manner creates a new path that is equivalent to an activator–inhibitor type of circuit that can generate oscillations (Fig. 5*C*). Oscillations can also emerge from two FFLs that interact with each other where one serves as an activator and the other as a repressor (Fig. 5*D*). This example demonstrates the importance of exploring the way network motifs are integrated in a large network in order to understand the potential properties that the network can exhibit. Although oscillations cannot arise from individual FFLs for any choice of parameter values (oscillations are impossible in any strictly feedforward motif), they can emerge when the FFLs mutually interact with each other in the large network.

## Discussion

Complex systems are composed of multiple levels of organization. Here, we developed a theoretical framework to identify and explore the intermediate levels of organization in complex networks. We defined how two subgraphs are joined together in a network at two levels by sharing at least one node or by being directly linked by at least one edge. We developed a method to reveal how network motifs are assembled into hypermotifs in real complex networks and demonstrated it in diverse evolved and designed networks. Finally, we used a nonlinear class of models to explore potential emergent properties of combinations and interactions of network motifs including the autoregulation, FFL, and several feedback circuits.

Applying the network hypermotif framework on real networks can help to reveal patterns in the inner topological structure of the network and to make specific predictions on the behavior that is expected to emerge at the mesoscale level of the network. Exploring properties of combinations of building-block circuits provides a way to rigorously explore emergence in complex systems and to define new levels of organization based on functional modules that provide important and emergent properties.

The method we present in this work to identify combinations of network motifs in real networks is not limited by the size of the motif and does not entail counting specific higher-order subgraphs, which can be computationally hard. However, in order to estimate the statistical significance of hypermotif circuits, one needs to randomize the network such that the frequencies of all subgraphs up to the size of the motifs considered is kept constant. Creating such a null model is currently feasible for up to three-node motifs. Future work can address larger motifs by developing a method of network randomization without affecting the frequencies of network motifs of any size.

Another challenge in the hypermotif framework is to identify the real functionality that is associated to the interacting motifs in different real-world networks. Here we demonstrated a variety of potential dynamic behavior that overrepresented hypermotif circuits can exhibit. Future work can focus on enriched hypermotif circuits in specific contexts and explore their functionality experimentally. In our modeling approach we focused on examining the dynamical behavior of hypermotifs in biological and ecological networks. It would be interesting to explore the properties of the hypermotifs that are enriched in other types of networks including social and linguistics networks which are represented by virtual links rather than physical links. It would be also interesting to further explore the role of simple larger motifs versus hypermotifs which are composed of smaller motifs in real networks.

Here, we considered how network motifs are joined in networks with pairwise interactions where edges connect a pair of nodes. Network motifs were previously identified also in hypergraphs where edges can connect any number of nodes describing, for example, collaborations of researchers and joint interactions of proteins (46). It would be interesting to generalize the hypermotif framework to hypergraphs where motifs can be joined through specific hypernodes in the network.

The framework presented here could be useful for developing a new mnemonic way to categorize the nodes in a network into distinct classes based on their position in the network’s motifs and their higher-order modules. This categorization is complement to previously suggested criteria including the nodes degree and contrabillity properties (47) where nodes with the same level of connectivity can play different roles in the network’s modules. Identifying these categories of nodes in real networks can highlight the key drivers of the network’s emergent properties. For example, in biology this could have important implications in pinpointing genes that are key for essential biological processes as well as genes with a potential to drive a biological system into a pathological state.

## Materials and Methods

### Counting All Possible Combinations and Interactions of Network Motifs.

Consider two network motifs, A and B, with $n_{A}$ and $n_{B}$ nodes, respectively. A and B can be combined by sharing $N_{v}$ nodes such that the following condition applies: $1\;\leq \;N_{v}<\min(n_{A},n_{B}).$ This condition ensures that the topology of each motif is kept as a subgraph of the combined circuit. The identity of the$\;N_{v}$ nodes that are shared among the two motifs defines a core topology of a combination of the two motifs. Each core topology can be extended to include additional edges between pairs of nodes that do not participate in the same motif (*SI Appendix*, Fig. S1*A*).

A and B interact when they have at least one edge that directly links them. Since each node from motif A can interact with each node from motif B, the maximal number of possible edges is the number of pairs of nodes that do not participate in the same motif, $n_{A}n_{B}$. For directed networks where every pair of nodes can have two directed edges this number should be multiplied by 2. The number of possible topologies of interaction between motifs A and B is therefore $2^{2n_{A}n_{B}}$ for directed networks and $2^{n_{A}n_{B}}$ for undirected networks (*SI Appendix*, Fig. S1*B*). We note that in both combinations and interactions of motifs there could be circuits that are isomorphic to each other and therefore the number of unique topologies may be smaller than the maximal number of circuit topologies.

### Detecting Enriched Combinations of Network Motifs in Real Networks.

Consider a network G with N nodes and E edges. To detect over- and underrepresented combinations of network motifs we followed the following steps:

1) Identify network motifs of up to three nodes (see remark below). We used MFinder (7) and also verified consistency with finding network motifs in Mathematica using the IGraphM package and its function IGRewire to randomize the network and IGMotifs to find the motifs and their frequencies. We denote $N_{m}$ as the set of nodes in G that participate in the network motifs.
2) Categorize nodes in $N_{m}$ to different groups according to their roles in the network motifs: $\left\{n_{1},n_{2},\ldots ,n_{k}\right\}$, where *k* is the number of the different network motif roles in G. Note that certain nodes in $N_{m}$ may appear in more than one of the $\left\{n_{1},n_{2},\ldots ,n_{k}\right\}$ groups (which are the cases where network motifs are combined).
3) Compute the Jaccard index for all k(k−1)/2 pairs of motif roles: $J(n_{i},n_{j})=|n_{i}\cap n_{j}|/|n_{i}\cup n_{j}|$, which is the size of the intersection of nodes in roles *i*,*j* divided by the size of their union.
4) For nodes that appear more than once in the same role, we compute the Jaccard index by the ratio of the number of nodes that appear in a network motif role more than once to the total number of nodes that participate in that motif role.
5) We use the MFinder package to create 100 random networks that have N nodes, E edges, the same incoming and outgoing edges per node, and the same frequency of all subgraphs up to three-node subgraphs.
6) We repeat steps 2 through 4 for all the random networks and compute $J_{\mathrm{rand}}(n_{i},n_{j})$ for all pairs of motif roles for the random networks.
7) For every *i*,*j* such that i,j∈{1,…,k} we compute the Z-score of $J(n_{i},n_{j})$ of network G from the distribution of $\left\{J_{\mathrm{rand}}(n_{i},n_{j})\right\}$:$Z_{ij}=(J(n_{i},n_{j})-\mathrm{mean}(\{J_{\mathrm{rand}}(n_{i},n_{j})\}))/\mathrm{std}(\{J_{\mathrm{rand}}(n_{i},n_{j})\}$ and compute the *P* value by estimating the cumulative density of $Z_{ij}$ for a normal distribution with zero mean and unit variance. We use the function NormalPValue in the package HypothesisTesting in Mathematica to compute the *P* value. We then use the Benjamini–Hochberg procedure to correct for multiple hypothesis testing, which provides us a corrected q-value for each pair of motif roles.
8) We consider over- and underrepresented motif combinations if their q-value is smaller than 0.05, where $Z_{ij}>0$ for overrepresented combinations and $Z_{ij}<0$ for underrepresented combinations.

Our method detects situations in which two network motifs are joined by sharing certain nodes. The difference between our method and detecting network motifs of a larger size is that our method does not detect a specific subgraph as a recurring pattern but rather finds classes of subgraphs in which the two network motifs in question are combined in a certain way. This means that in certain networks the exact topology of the combination of the two network motifs may include additional edges that are in accordance with our definition of a core topology of a combination of network motifs and its possible extensions (*SI Appendix*, Fig. S1*A*). In *SI Appendix*, Fig. S3*A* we show the frequencies of the core topologies of each overrepresented motif combination and its extensions. We find that in most cases the most enriched motif combinations are with a core topology (without additional edges).

To analyze the word adjacency network, we downsampled the network (G) using the following steps:

1) Define the size of the downsampling, sz, as a parameter that can be tuned.
2) We construct a list of nodes which we sample, s, from the large network G.
3) Randomly choose one node from G, $s_{0}$, to be the first entry of s.
4) Randomly choose a node that is a member of the neighborhood of $s_{0}$, $s_{1}\in N_{s_{0}}$, to be the next entry of s.
5) Insert additional entries to s for i∈{2,…,sz/3} by randomly choosing a node that is a member of the neighborhood of $s_{i-1}$ with probability of 85%, or a node that is a member of the neighborhood of $s_{0}$.
6) Certain entries of s could be repeated more than once. If the length of the unique list of s is smaller than (sz/3)/2 consider a new $s_{0}$ which is a randomly chosen node from G. Otherwise, keep the previous $s_{0}$.
7) Insert additional entries to s for i∈{sz/3+1,…,2sz/3} by randomly choosing a node that is a member of the neighborhood of $s_{i-1}$ with probability of 85%, or a node that is a member of the neighborhood of $s_{0}$.
8) If the length of the unique list of s is smaller than (sz/3) consider a new $s_{0}$ which is a randomly chosen node from G. Otherwise, keep the previous $s_{0}$.
9) Insert additional entries to s for i∈{2sz/3+1,…,sz} by randomly choosing a node that is a member of the neighborhood of $s_{i-1}$ with probability of 85%, or a node that is a member of the neighborhood of $s_{0}$.
10) The downsampled network $G_{d}$ is the network that contains nodes from the unique list of s.

We repeated the downsampling procedure multiple times with varying sampling size (sz), where we check that the downsampled networks show a similar degree distribution and the same network motifs as the complete large network.

### Modeling Known Network Motifs and Their Combinations.

We model several previously identified network motifs where we consider that each node has a linear removal term and that interactions between nodes can be described by Hill functions. A positive interaction from node X to node Y is modeled by an increasing Hill function, $Y^{n_{xy}}/(k_{xy}{}^{n_{xy}}+Y^{n_{xy}})$, and a negative interaction is modeled by a decreasing Hill function, $k_{xy}{}^{n_{xy}}/(k_{xy}{}^{n_{xy}}+Y^{n_{xy}})$. Each interaction is therefore described by two parameters: the cooperativity coefficient $n_{xy}$ and the level at which the interaction effect reaches halfway of its maximal level $k_{xy}$. We used the functions Streamplot and Contourplot in Mathematica 12.1.1.0 to plot the phase portraits of the circuits.

The models and parameter values that we use in Fig. 3 are listed in *SI Appendix*.

In *SI Appendix* we explore combinations of FFL circuits with self-loops on the different FFL nodes where we assume that X is not a dynamical variable but rather rises in a step function manner. This assumption allows us to explore the phase portraits of Y and Z and to compare their nullclines when the self-loop appears on Y or Z (*SI Appendix*, Fig. S4 *A* and *B*).

### Modeling Enriched Combinations in Real Networks.

The models and parameter values that we use to model several observed overrepresented combinations of network motifs are listed in *SI Appendix*.

In the combination of two three-node loop circuits (Fig. 4*C*), undamped oscillations emerge in the all-positive-interactions three-node loop circuit. The reason is that pure oscillations require negative feedback and a delay. However, we note that this all-positive-interactions circuit can show damped oscillations (with a spiral fixed point) which in the presence of noise can become undamped oscillations (11).

### Modeling Known Network Motifs and Their Interactions.

The models and parameter values that we use to model interactions of network motifs that are shown in Fig. 5 are listed in *SI Appendix*.

In the example of an interaction between two FFLs (Fig. 5*D*), the oscillations are an emergent property since they are absent from FFL circuits on their own. To prove that, consider the Jacobian matrix of the FFL circuit: $J=\left(\begin{array}{ccc} -1 & 0 & 0\\ 1 & -1 & 0\\ 1 & 1 & -1 \end{array}\right)$, which is a lower triangular matrix. The eigenvalues of a lower (or an upper) triangular matrix are the entries on its diagonal and therefore they are always −1 in our case and cannot be complex.

## Supplementary Material

Supplementary File

## Data Availability

The code and data files (48) required to generate all the figures in this study have been deposited in a publicly accessible database and are available on GitHub at https://github.com/miriadler/network-hyper-motifs. All other study data are included in the article and/or *SI Appendix*.
