# Supplementary material for: Emergence of dynamic properties in network hypermotifs
Source: Proc Natl Acad Sci U S A. 2022 Aug 1;119(32):e2204967119. doi: 10.1073/pnas.2204967119 (PMC9371713; doi:10.1073/pnas.2204967119)
Supplement: Supplementary File [file pnas.2204967119.sapp.pdf]

Supplementary Information for:

Emergence of dynamic properties in network hyper-motifs

Miri Adler[1] and Ruslan Medzhitov[2,*]

[1]Broad Institute of Massachusetts Institute of Technology and Harvard, Cambridge, MA 02142, USA

[2]Howard Hughes Medical Institute, Department of Immunobiology, Yale University School of Medicine, New Haven, CT 06510, USA

*Corresponding author: Ruslan Medzhitov

Email: ruslan.medzhitov@yale.edu

SI Figures

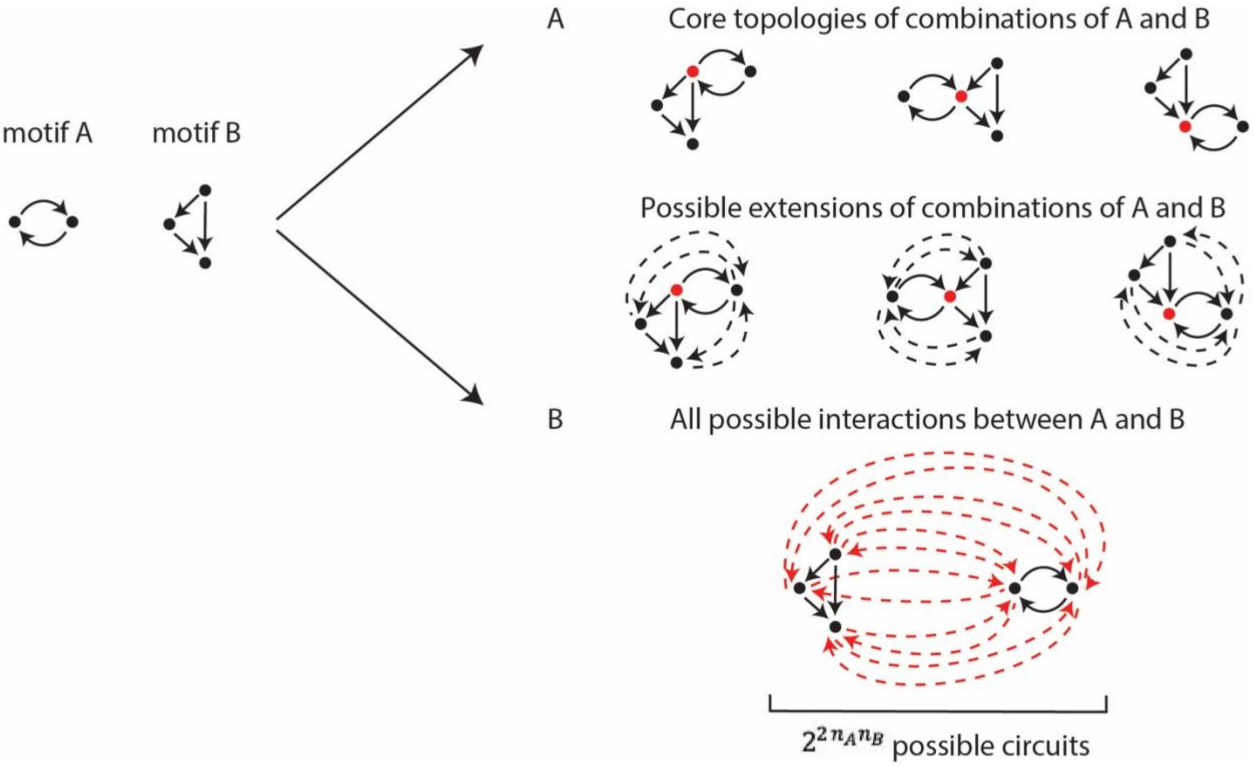

Figure S1: A. Definition of core topologies of combinations of motifs A and B (where they simply share at least one node), and their possible extensions in which every pair of nodes that do not participate in the same motif can be linked, exemplified for the feedback and feedforward loop motifs. The shared nodes are marked in red and the dashed edges represent the possible added links in the possible extensions. In this example there are 16 different topologies for each core combination. **B.** Example of all possible interactions between a feedback and a feedforward loop where every pair of nodes from the different motifs can interact with each other (marked in dashed red), amounting to $2^{2n_A n_B}$possible topologies for directed networks.

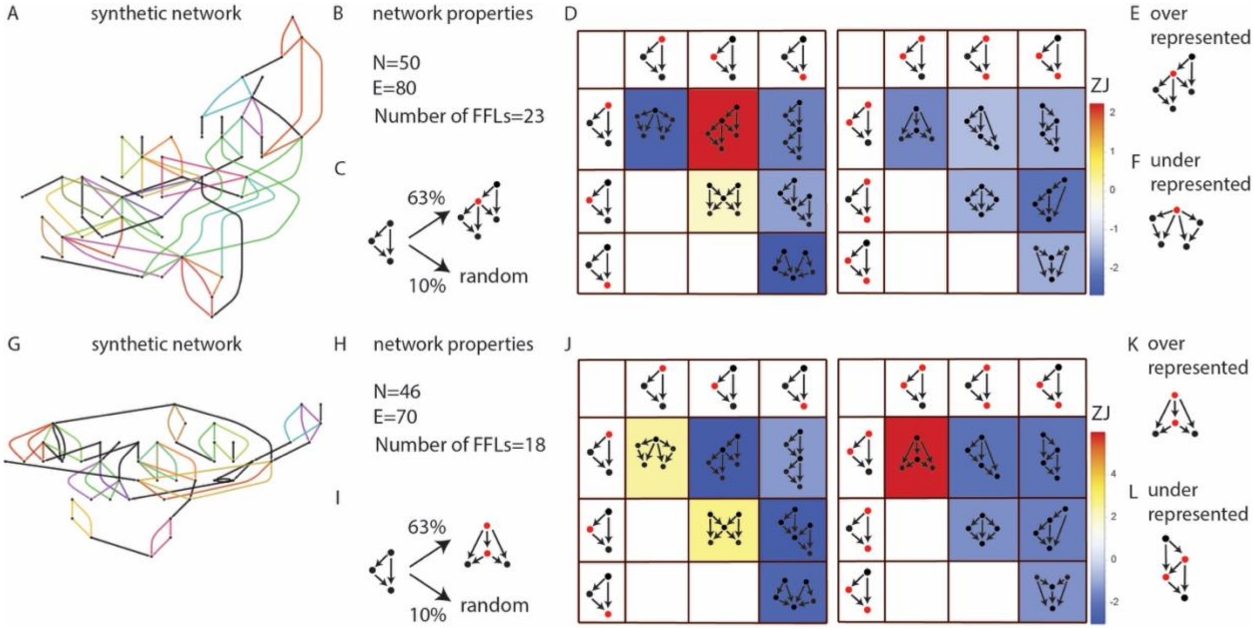

Figure S2: A. A synthetic network in which the FFLs edges are colored and edges that do not participate in an FFL are in black. **B.** properties of the network in A. **C.** The network was generated where 63% of all possible FFL pairs were combined in the specified pattern, and 10% were combined randomly. **D.** Results of our method to detect enriched combinations of network motifs. The hyper-motifs are colored based on the standard deviation of its Jaccard index (ZJ). **E-F** Over-represented (E) and under-represented (F) motif combinations of the synthetic network in A. **G-L** The same as in A-F only for a different synthetic network.

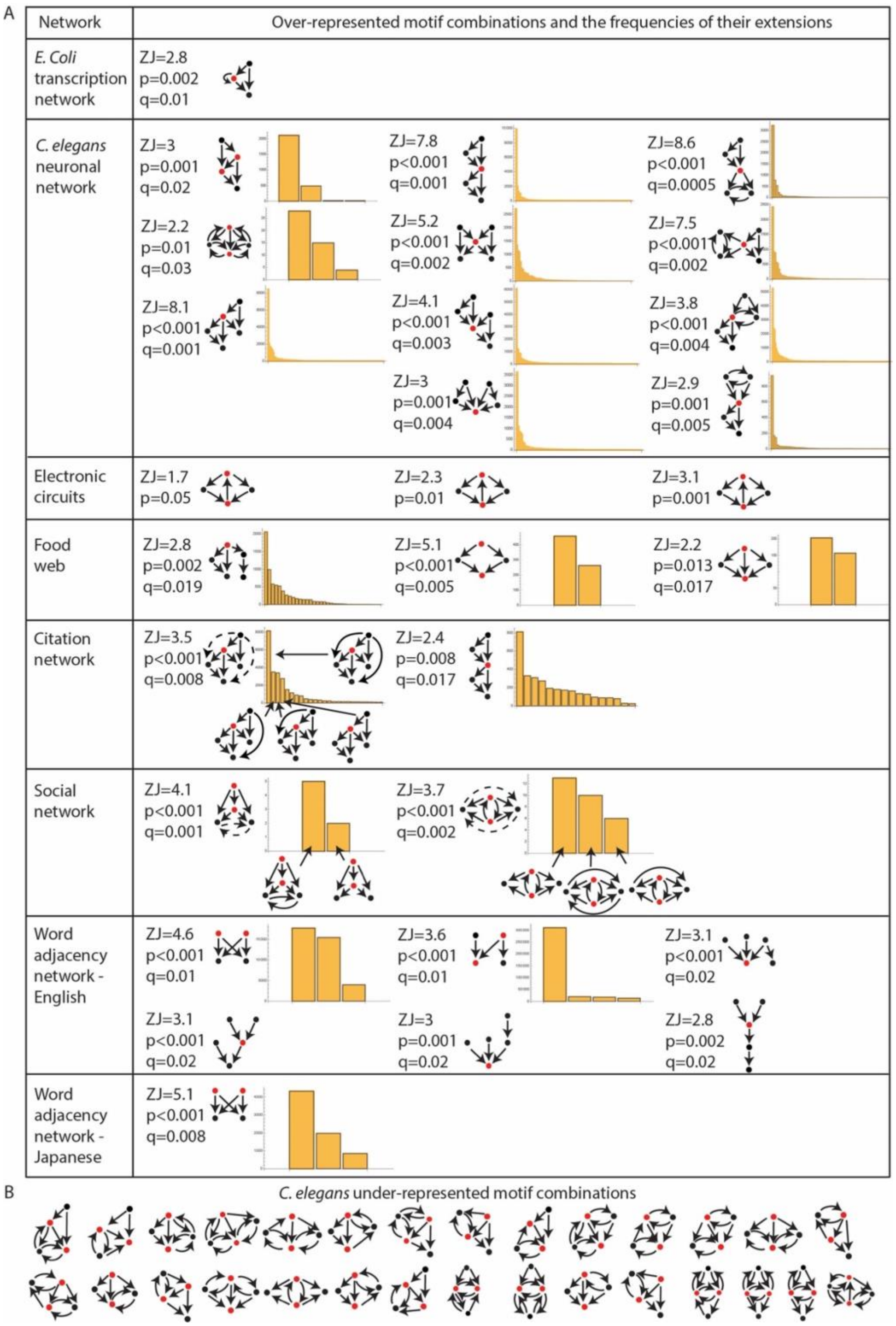

Figure S3: A. A table with the networks analyzed in the main text and their over-represented motif combinations. For each over-represented motif combination, we show the standard deviation of its Jaccard index (ZJ), its p-value (p) and the adjusted q-value (q). We also plot for each core topology of enriched motif combination a histogram for the frequencies of the core topology and its possible extensions in the network. Unless specified otherwise, the most frequent

topology is the core topology of the motif combinations. **B.** Topologies of additional under-represented motif combinations of the *C. elegans* neuronal network.

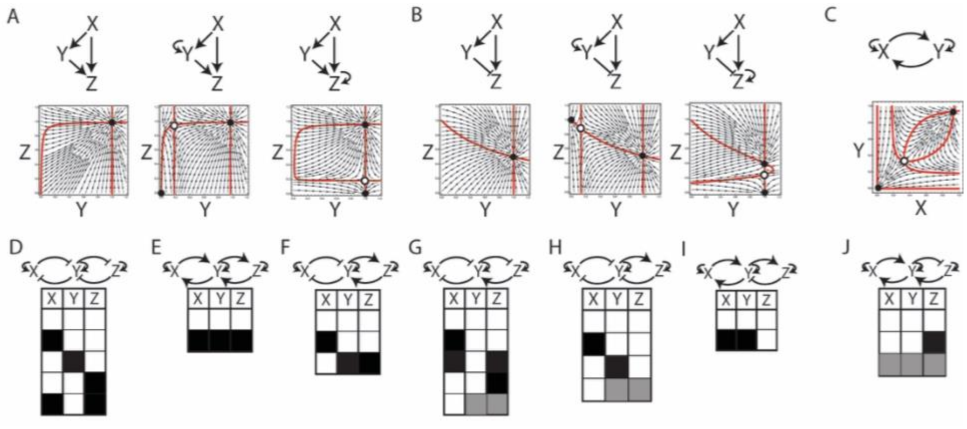

Figure S4: A-B. Phase portraits of the type 1 coherent (A) and incoherent (B) FFL with no autoregulation, and with a positive autoregulation of the intermediate node Y or the output node Z. **C.** Phase portrait of the lock-ON circuit in which both X and Y positively autoregulate their own levels. **D-J.** Combinations of two feedback circuits and their possible steady state where a white square represents the OFF state, black square represents an ON state, and a gray square represents an intermediate state with damped oscillations.

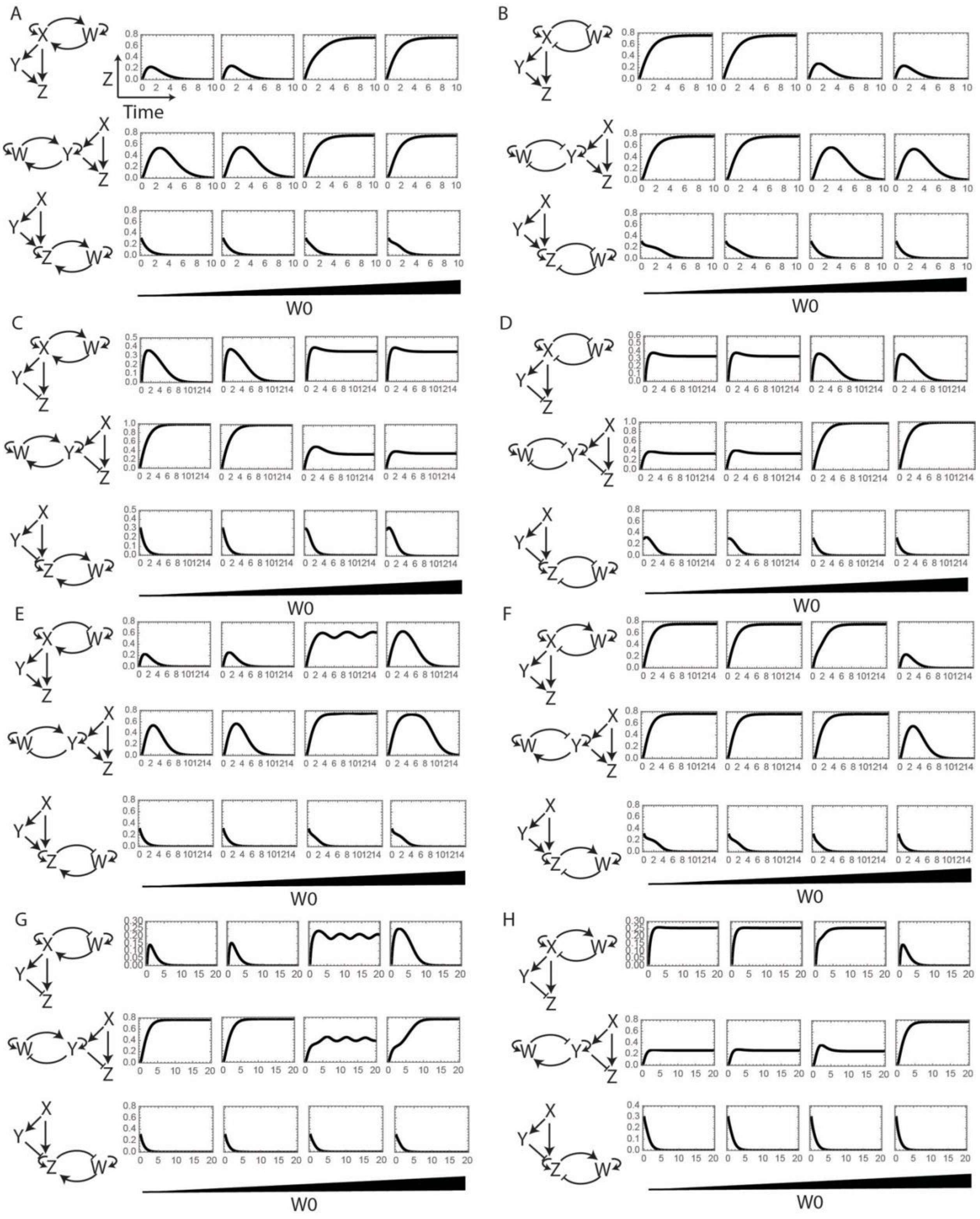

Figure S5: A-H. All possible core topologies of combinations of a feedforward loop and a feedback circuit where the dynamics of the FFL's output node, Z, is plotted for varying initial conditions of the node that participate only in the feedback circuit, W. Details about models and parameters are in the SI.

Supplementary Information

Generating synthetic network to test our method to detect enriched hyper-motifs

We generated two synthetic networks with specified distributions of network motifs and network hyper-motifs and applied our method to detect statistically enriched combinations of network motifs to test the method.
To generate the networks, we first considered 20 autonomous FFLs making up an unconnected network with 60 nodes and 60 edges. We then randomly selected 63% out of all possible 190 pairs of FFLs and combined them into a particular combination. We also randomly selected 10% out of all possible 190 pairs of FFLs and randomly combined them into one of the 12 possible combinations of two FFLs. This procedure slightly reduced the number of nodes in the synthetic network since some of them were fused into one shared node. We then applied our method to detect enriched combinations of network motifs (described in Figure 2A) and found that the most enriched hyper-motifs are in line with the hyper-motifs we combined the FFLs into. Interestingly, the synthetic networks show certain under-represented combinations of motifs that unintentionally emerged from the way the networks were generated (Fig S2F, L).

Models and parameters used in Figure 3

We list the models and parameter values that we use in Figure 3:

- Positive self-loop (Fig 3A):
  1. $X' = X^{n_{xx}}/({k_{xx}}^{n_{xx}} + X^{n_{xx}}) - X$

The cooperativity, n_{xx}, has to be at least 2 for this model of a self-loop circuit to show bistability.

- Toggle-switch feedback circuit (Fig 3B):
  2. $X' = {k_{yx}}^{n_{yx}}/({k_{yx}}^{n_{yx}} + Y^{n_{yx}}) - X$
  3. $Y' = {k_{xy}}^{n_{xy}}/({k_{xy}}^{n_{xy}} + X^{n_{xy}}) - Y$
- Lock-ON feedback circuit (Fig 3C):
  4. $X' = Y^{n_{yx}}/({k_{yx}}^{n_{yx}} + Y^{n_{yx}}) - X$
  5. $Y' = X^{n_{xy}}/({k_{xy}}^{n_{xy}} + X^{n_{xy}}) - Y$
- Oscillator feedback circuit (Fig 3D):
  6. $X' = Y^{n_{yx}}/({k_{yx}}^{n_{yx}} + Y^{n_{yx}}) - X$
  7. $Y' = {k_{xy}}^{n_{xy}}/({k_{xy}}^{n_{xy}} + X^{n_{xy}}) - Y$

The phase portraits for the feedback circuits in Figure 3A-D are plotted using the above models (eqs. 2-7) with $n_{yx} = n_{xy} = 3, k_{yx} = k_{xy} = 0.3$.

- Combination of positive self-loops and a toggle-switch circuit (Fig 3G):
  8. $X' = (X^{n_{xx}}/({k_{xx}}^{n_{xx}} + X^{n_{xx}}))({k_{yx}}^{n_{yx}}/({k_{yx}}^{n_{yx}} + Y^{n_{yx}})) - X,\ n_{xx} = n_{yx} = 3, k_{xx} = k_{yx} = 0.3$
  9. $Y' = (Y^{n_{yy}}/({k_{yy}}^{n_{yy}} + Y^{n_{yy}}))({k_{xy}}^{n_{xy}}/({k_{xy}}^{n_{xy}} + X^{n_{xy}})) - Y, n_{yy} = n_{xy} = 3, k_{yy} = k_{xy} = 0.3$
- Combination of positive self-loops and an oscillator circuit (Fig 3H):
  10. $X' = (X^{n_{xx}}/({k_{xx}}^{n_{xx}} + X^{n_{xx}}))(Y^{n_{yx}}/({k_{yx}}^{n_{yx}} + Y^{n_{yx}})) - X,\ n_{xx} = n_{yx} = 3, k_{xx} = 0.2, k_{yx} = 0.3$
  11. $Y' = (Y^{n_{yy}}/({k_{yy}}^{n_{yy}} + Y^{n_{yy}}))({k_{xy}}^{n_{xy}}/({k_{xy}}^{n_{xy}} + X^{n_{xy}})) - Y, n_{yy} = n_{xy} = 3, k_{yy} = 0.2, k_{xy} = 0.3$
- Combination of a positive self-loop and feedforward loop (FFL) circuits:
  The model for a coherent FFL (Fig 3I):

12. $X' = 1 - X$
13. $Y' = X^{n_{xy}}/({k_{xy}}^{n_{xy}} + X^{n_{xy}}) - Y,\ n_{xy} = 1, k_{xy} = 0.01$
14. $Z' = (X^{n_{xz}}/({k_{xz}}^{n_{xz}} + X^{n_{xz}}))(Y^{n_{yz}}/({k_{yz}}^{n_{yz}} + Y^{n_{yz}})) - Z,\ n_{xz} = n_{yz} = 1, k_{xz} = k_{yz} = 0.01$

The model for an incoherent FFL (Fig 3J):

15. $X' = 1 - X$
16. $Y' = X^{n_{xy}}/({k_{xy}}^{n_{xy}} + X^{n_{xy}}) - Y,\ n_{xy} = 1, k_{xy} = 0.01$
17. $Z' = (X^{n_{xz}}/({k_{xz}}^{n_{xz}} + X^{n_{xz}}))({k_{yz}}^{n_{yz}}/({k_{yz}}^{n_{yz}} + Y^{n_{yz}})) - Z,\ n_{xz} = n_{yz} = 1, k_{xz} = 0.01, k_{yz} = 0.5$

To combine the FFL circuits with a self-loop on one of its nodes, i, we multiply the production term of variable i by the following term: $(i^{n_{ii}}/({k_{ii}}^{n_{ii}} + i^{n_{ii}})), n_{ii} = 3, k_{ii} = 0.3$ in the coherent FFL and $k_{xx} = k_{yy} = 0.3, k_{zz} = 0.15$ in the incoherent FFL.

- Combinations of two oscillator circuits:

First combination (Fig 3K):

18. $X' = (X^{n_{xx}}/({k_{xx}}^{n_{xx}} + X^{n_{xx}}))(Y^{n_{yx}}/({k_{yx}}^{n_{yx}} + Y^{n_{yx}})) - X,\ n_{xx} = n_{yx} = 3, k_{xx} = 0.2, k_{yx} = 0.3$
19. $Y' = (Y^{n_{yy}}/({k_{yy}}^{n_{yy}} + Y^{n_{yy}}))({k_{xy}}^{n_{xy}}/({k_{xy}}^{n_{xy}} + X^{n_{xy}}))(Z^{n_{zy}}/({k_{zy}}^{n_{zy}} + Z^{n_{zy}})) - Y, n_{yy} = n_{xy} = n_{zy} = 3, k_{yy} = 0.2, k_{xy} = k_{zy} = 0.3$
20. $Z' = (Z^{n_{zz}}/({k_{zz}}^{n_{zz}} + Z^{n_{zz}}))({k_{yz}}^{n_{yz}}/({k_{yz}}^{n_{yz}} + Y^{n_{yz}})) - Z,\ n_{zz} = n_{yz} = 3, k_{zz} = 0.2, k_{yz} = 0.3$

Second combination (Fig 3L):

21. $X' = (X^{n_{xx}}/({k_{xx}}^{n_{xx}} + X^{n_{xx}}))(Y^{n_{yx}}/({k_{yx}}^{n_{yx}} + Y^{n_{yx}})) - X,\ n_{xx} = n_{yx} = 3, k_{xx} = 0.2, k_{yx} = 0.3$
22. $Y' = (Y^{n_{yy}}/({k_{yy}}^{n_{yy}} + Y^{n_{yy}}))({k_{xy}}^{n_{xy}}/({k_{xy}}^{n_{xy}} + X^{n_{xy}}))({k_{zy}}^{n_{zy}}/({k_{zy}}^{n_{zy}} + Z^{n_{zy}})) - Y$
$n_{yy} = n_{xy} = n_{zy} = 3, k_{yy} = 0.2, k_{xy} = k_{zy} = 0.3$
23. $Z' = (Z^{n_{zz}}/({k_{zz}}^{n_{zz}} + Z^{n_{zz}}))(Y^{n_{yz}}/({k_{yz}}^{n_{yz}} + Y^{n_{yz}})) - Z,\ n_{zz} = n_{yz} = 3, k_{zz} = 0.2, k_{yz} = 0.3$

Third combination (Fig 3M):

24. $X' = (X^{n_{xx}}/({k_{xx}}^{n_{xx}} + X^{n_{xx}}))({k_{yx}}^{n_{yx}}/({k_{yx}}^{n_{yx}} + Y^{n_{yx}})) - X,\ n_{xx} = n_{yx} = 3, k_{xx} = 0.2, k_{yx} = 0.3$
25. $Y' = (Y^{n_{yy}}/({k_{yy}}^{n_{yy}} + Y^{n_{yy}}))(X^{n_{xy}}/({k_{xy}}^{n_{xy}} + X^{n_{xy}}))(Z^{n_{zy}}/({k_{zy}}^{n_{zy}} + Z^{n_{zy}})) - Y$
$n_{yy} = n_{xy} = n_{zy} = 3, k_{yy} = 0.2, k_{xy} = k_{zy} = 0.3$
26. $Z' = (Z^{n_{zz}}/({k_{zz}}^{n_{zz}} + Z^{n_{zz}}))({k_{yz}}^{n_{yz}}/({k_{yz}}^{n_{yz}} + Y^{n_{yz}})) - Z,\ n_{zz} = n_{yz} = 3, k_{zz} = 0.2, k_{yz} = 0.3$

- Combination of an oscillator circuit and a coherent FFL through the FFL's intermediate node (Fig 3N):

27. $X' = 1 - X$
28. $Y' = (Y^{n_{yy}}/({k_{yy}}^{n_{yy}} + Y^{n_{yy}}))(X^{n_{xy}}/({k_{xy}}^{n_{xy}} + X^{n_{xy}}))(W^{n_{wy}}/({k_{wy}}^{n_{wy}} + W^{n_{wy}})) - Y$
$n_{yy} = n_{wy} = 3, n_{xy} = 1, k_{yy} = 0.2, k_{wy} = 0.3, k_{xy} = 0.01$
29. $Z' = (X^{n_{xz}}/({k_{xz}}^{n_{xz}} + X^{n_{xz}}))(Y^{n_{yz}}/({k_{yz}}^{n_{yz}} + Y^{n_{yz}})) - Z,\ n_{xz} = n_{yz} = 1, k_{xz} = k_{yz} = 0.01$
30. $W' = (W^{n_{ww}}/({k_{ww}}^{n_{ww}} + W^{n_{ww}}))({k_{yw}}^{n_{yw}}/({k_{yw}}^{n_{yw}} + Y^{n_{yw}})) - W,\ n_{ww} = n_{yw} = 3, k_{ww} = 0.2, k_{yw} = 0.3$

- Combination of an oscillator circuit and an incoherent FFL through the FFL's intermediate node (Fig 3O):

31. $X' = 1 - X$

32. $Y' = (Y^{n_{yy}}/({k_{yy}}^{n_{yy}} + Y^{n_{yy}}))(X^{n_{xy}}/({k_{xy}}^{n_{xy}} + X^{n_{xy}}))(W^{n_{wy}}/({k_{wy}}^{n_{wy}} + W^{n_{wy}})) - Y$

$n_{yy} = n_{wy} = 3, n_{xy} = 1, k_{yy} = 0.2, k_{wy} = 0.3, k_{xy} = 0.01$

33. $Z' = (X^{n_{xz}}/({k_{xz}}^{n_{xz}} + X^{n_{xz}}))({k_{yz}}^{n_{yz}}/({k_{yz}}^{n_{yz}} + Y^{n_{yz}})) - Z,\ n_{xz} = n_{yz} = 1, k_{xz} = 0.01, k_{yz} = 0.5$

34. $W' = (W^{n_{ww}}/({k_{ww}}^{n_{ww}} + W^{n_{ww}}))({k_{yw}}^{n_{yw}}/({k_{yw}}^{n_{yw}} + Y^{n_{yw}})) - W,\ n_{ww} = n_{yw} = 3, k_{ww} = 0.2, k_{yw} = 0.3$

We plot the dynamical behavior of all possible combinations between these network motifs for a given choice of model parameters (Fig. S4C-J, S5).

Modeling combinations of feedforward loop circuits and autoregulation as a two-dimensional system

To better characterize the differences between FFL circuits in which the input, intermediate or the output nodes autoregulate their own levels, we consider a model in which the input variable (X) is not a dynamical variable but rather assume that it rises in a step function manner at t=0 to its final level, X_f. This assumption allows us to model these circuits with only two variables, Y and Z, and thus to plot their two-dimensional phase portraits (fig. S4A-B). The model we use for combinations of a coherent FFL and positive autoregulation is therefore:

35. $Y' = X_f - Y$

36. $Z' = X_f\ Y^{n_{yz}}/(Y^{n_{yz}} + {k_{yz}}^{n_{yz}}) - Z$

To combine the FFL circuit with autoregulation we multiply the production term of Y or Z with $Y^{n_{yy}}/(Y^{n_{yy}} + {k_{yy}}^{n_{yy}})$or $Z^{n_{zz}}/(Z^{n_{zz}} + {k_{zz}}^{n_{zz}})$. In Figure S3A we used $X_f = 1, k_{yz} = 0.01, k_{yy} = k_{zz} = 0.3, n_{yz} = 1, n_{yy} = n_{zz} = 3$.

The model for combinations of an incoherent FFL and positive autoregulation:

37. $Y' = X_f - Y$

38. $Z' = X_f\ Y^{n_{yz}}/({k_{yz}}^{n_{yz}} + {k_{yz}}^{n_{yz}}) - Z$

And we combine autoregulation in the same way as described for the coherent FFL circuits. In Figure S3B we used $X_f = 1, k_{yz} = 1, k_{yy} = k_{zz} = 0.25, n_{yz} = 1, n_{yy} = n_{zz} = 3$

Models and parameters used in Figure 4

We now list the models and parameter values that we use to model several observed over-represented combinations of network motifs that are shown in Figure 2:

- Combination of two double mutual feedback circuits where all interactions are positive (Fig. 4A):

  39. $X' = (W^{n_{wx}}/({k_{wx}}^{n_{wx}} + W^{n_{wx}}))(Y^{n_{yx}}/({k_{yx}}^{n_{yx}} + Y^{n_{yx}})) - X$

  40. $Y' = (Z^{n_{zy}}/({k_{zy}}^{n_{zy}} + Z^{n_{zy}}))(X^{n_{xy}}/({k_{xy}}^{n_{xy}} + X^{n_{xy}})) - Y$

  41. $Z' = (X^{n_{xz}}/({k_{xz}}^{n_{xz}} + X^{n_{xz}}))(Y^{n_{yz}}/({k_{yz}}^{n_{yz}} + Y^{n_{yz}}))(W^{n_{wz}}/({k_{wz}}^{n_{wz}} + W^{n_{wz}})) - Z$

  42. $W' = (X^{n_{xw}}/({k_{xw}}^{n_{xw}} + X^{n_{xw}}))(Z^{n_{zw}}/({k_{zw}}^{n_{zw}} + Z^{n_{zw}})) - W$

  Where we used the following parameter values:

  $k_{wx} = k_{xw} = k_{xy} = k_{yx} = k_{xz} = k_{yz} = k_{zy} = 0.01, k_{wz} = 3.38, k_{zw} = 0.16, n_{ij} = 3$

- Combination of two double mutual feedback circuits where all interactions are positive except for the edge between X and Z where X inhibits Z (Fig. 4B):

  43. $X' = (W^{n_{wx}}/({k_{wx}}^{n_{wx}} + W^{n_{wx}}))(Y^{n_{yx}}/({k_{yx}}^{n_{yx}} + Y^{n_{yx}})) - X$

  44. $Y' = (Z^{n_{zy}}/({k_{zy}}^{n_{zy}} + Z^{n_{zy}}))(X^{n_{xy}}/({k_{xy}}^{n_{xy}} + X^{n_{xy}})) - Y$

  45. $Z' = ({k_{xz}}^{n_{xz}}/({k_{xz}}^{n_{xz}} + X^{n_{xz}}))(Y^{n_{yz}}/({k_{yz}}^{n_{yz}} + Y^{n_{yz}}))(W^{n_{wz}}/({k_{wz}}^{n_{wz}} + W^{n_{wz}})) - Z$

  46. $W' = (X^{n_{xw}}/({k_{xw}}^{n_{xw}} + X^{n_{xw}}))(Z^{n_{zw}}/({k_{zw}}^{n_{zw}} + Z^{n_{zw}})) - W$

  Where we used the following parameter values:

$k_{wx} = k_{xw} = k_{xy} = k_{xz} = k_{yz} = k_{zy} = k_{wz} = k_{zw} = 0.01, k_{yx} = 1.87$, and the cooperativity parameters are $n_{ij} = 3$

- Combination of two 3-node loop circuits where all interactions are positive except for the edge between Y and Z where Y inhibits Z (Fig. 4C):
  47. $X' = (W^{n_{wx}}/({k_{wx}}^{n_{wx}} + W^{n_{wx}}))(Z^{n_{zx}}/({k_{zx}}^{n_{zx}} + Z^{n_{zx}})) - X$
  48. $Y' = (X^{n_{xy}}/({k_{xy}}^{n_{xy}} + X^{n_{xy}})) - Y$
  49. $Z' = ({k_{yz}}^{n_{yz}}/({k_{yz}}^{n_{yz}} + Y^{n_{yz}})) - Z$
  50. $W' = (Y^{n_{yw}}/({k_{yw}}^{n_{yw}} + Y^{n_{yw}})) - W$

  Where we used the following parameter values:
  $k_{wx} = k_{xy} = k_{yw} = k_{yz} = 0.01, k_{zx} = 4.7$, and the cooperativity parameters are $n_{ij} = 3$

Models and parameters used in Figure 5

We list the models and parameter values that we use to model interactions of network motifs that are shown in Figure 5:

- Interaction between two oscillator circuits in a toggle switch topology (Fig. 5A, option #1):
  51. $X' = (X^{n_{xx}}/({k_{xx}}^{n_{xx}} + X^{n_{xx}}))({k_{yx}}^{n_{yx}}/({k_{yx}}^{n_{yx}} + Y^{n_{yx}}))({k_{vx}}^{n_{vx}}/({k_{vx}}^{n_{vx}} + V^{n_{vx}})) - X$
  52. $Y' = (Y^{n_{yy}}/({k_{yy}}^{n_{yy}} + Y^{n_{yy}}))(X^{n_{xy}}/({k_{xy}}^{n_{xy}} + X^{n_{xy}})) - Y$
  53. $U' = (U^{n_{uu}}/({k_{uu}}^{n_{uu}} + U^{n_{uu}}))({k_{yu}}^{n_{yu}}/({k_{yu}}^{n_{yu}} + Y^{n_{yu}}))({k_{vu}}^{n_{vu}}/({k_{vu}}^{n_{vu}} + V^{n_{vu}})) - U$
  54. $V' = (V^{n_{vv}}/({k_{vv}}^{n_{vv}} + V^{n_{vv}}))(U^{n_{uv}}/({k_{uv}}^{n_{uv}} + U^{n_{uv}})) - V$

  Where we used the following parameter values:

$k_{ii} = 0.2, n_{ii} = 3, k_{xy} = k_{yx} = k_{uv} = k_{vu} = 0.3, k_{yu} = k_{vx} = 0.01, n_{xy} = n_{yx} = n_{uv} = n_{vu} = 3, n_{yu} = n_{vx} = 1$

- Interaction between two oscillator circuits in a toggle switch topology (Fig. 5A, option #2):
  55. $X' = (X^{n_{xx}}/({k_{xx}}^{n_{xx}} + X^{n_{xx}}))({k_{yx}}^{n_{yx}}/({k_{yx}}^{n_{yx}} + Y^{n_{yx}}))) - X$
  56. $Y' = (Y^{n_{yy}}/({k_{yy}}^{n_{yy}} + Y^{n_{yy}}))(X^{n_{xy}}/({k_{xy}}^{n_{xy}} + X^{n_{xy}}))({k_{uy}}^{n_{uy}}/({k_{uy}}^{n_{uy}} + U^{n_{uy}})) - Y$
  57. $U' = (U^{n_{uu}}/({k_{uu}}^{n_{uu}} + U^{n_{uu}}))({k_{vu}}^{n_{vu}}/({k_{vu}}^{n_{vu}} + V^{n_{vu}})) - U$
  58. $V' = (V^{n_{vv}}/({k_{vv}}^{n_{vv}} + V^{n_{vv}}))(U^{n_{uv}}/({k_{uv}}^{n_{uv}} + U^{n_{uv}}))({k_{xv}}^{n_{xv}}/({k_{xv}}^{n_{xv}} + X^{n_{xv}}) - V$

  Where we used the following parameter values:

$k_{ii} = 0.2, n_{ii} = 3, k_{xy} = k_{yx} = k_{uv} = k_{vu} = 0.3, k_{uy} = k_{xv} = 0.01, n_{xy} = n_{yx} = n_{uv} = n_{vu} = 3, n_{uy} = n_{xv} = 1$

- Interaction between two oscillator circuits in a lock-ON topology (Fig. 5B, option #1):
  59. $X' = (X^{n_{xx}}/({k_{xx}}^{n_{xx}} + X^{n_{xx}}))({k_{yx}}^{n_{yx}}/({k_{yx}}^{n_{yx}} + Y^{n_{yx}}))) - X$
  60. $Y' = (Y^{n_{yy}}/({k_{yy}}^{n_{yy}} + Y^{n_{yy}}))(X^{n_{xy}}/({k_{xy}}^{n_{xy}} + X^{n_{xy}}))(U^{n_{uy}}/({k_{uy}}^{n_{uy}} + U^{n_{uy}})) - Y$
  61. $U' = (U^{n_{uu}}/({k_{uu}}^{n_{uu}} + U^{n_{uu}}))({k_{vu}}^{n_{vu}}/({k_{vu}}^{n_{vu}} + V^{n_{vu}})) - U$
  62. $V' = (V^{n_{vv}}/({k_{vv}}^{n_{vv}} + V^{n_{vv}}))(U^{n_{uv}}/({k_{uv}}^{n_{uv}} + U^{n_{uv}}))(X^{n_{xv}}/({k_{xv}}^{n_{xv}} + X^{n_{xv}}) - V$

  Where we used the following parameter values:

$k_{ii} = 0.2, n_{ii} = 3, k_{xy} = k_{yx} = k_{uv} = k_{vu} = 0.3, k_{yu} = k_{vx} = 0.01, n_{xy} = n_{yx} = n_{uv} = n_{vu} = 3, n_{yu} = n_{vx} = 1$

- Interaction between two oscillator circuits in a lock-ON topology (Fig. 5B, option #2):
  63. $X' = (X^{n_{xx}}/({k_{xx}}^{n_{xx}} + X^{n_{xx}}))({k_{yx}}^{n_{yx}}/({k_{yx}}^{n_{yx}} + Y^{n_{yx}}))(V^{n_{vx}}/({k_{vx}}^{n_{vx}} + V^{n_{vx}})) - X$
  64. $Y' = (Y^{n_{yy}}/({k_{yy}}^{n_{yy}} + Y^{n_{yy}}))(X^{n_{xy}}/({k_{xy}}^{n_{xy}} + X^{n_{xy}})) - Y$
  65. $U' = (U^{n_{uu}}/({k_{uu}}^{n_{uu}} + U^{n_{uu}}))(Y^{n_{yu}}/({k_{yu}}^{n_{yu}} + Y^{n_{yu}}))({k_{vu}}^{n_{vu}}/({k_{vu}}^{n_{vu}} + V^{n_{vu}})) - U$
  66. $V' = (V^{n_{vv}}/({k_{vv}}^{n_{vv}} + V^{n_{vv}}))(U^{n_{uv}}/({k_{uv}}^{n_{uv}} + U^{n_{uv}})) - V$

  Where we used the following parameter values:

$k_{ii} = 0.2, n_{ii} = 3, k_{xy} = k_{yx} = k_{uv} = k_{vu} = 0.3, k_{uy} = k_{xv} = 0.01, n_{xy} = n_{yx} = n_{uv} = n_{vu} = 3, n_{uy} = n_{xv} = 1$

- Interaction between a coherent FFL and a toggle switch circuit (Fig. 5C):
  67. $X' = (V^{n_{vx}}/(k_{vx}{}^{n_{vx}} + V^{n_{vx}})) - X$
  68. $Y' = (X^{n_{xy}}/(k_{xy}{}^{n_{xy}} + X^{n_{xy}})) - Y$
  69. $Z' = (Y^{n_{yz}}/(k_{yz}{}^{n_{yz}} + Y^{n_{yz}}))(X^{n_{xz}}/(k_{xz}{}^{n_{xz}} + X^{n_{xz}})) - Z$
  70. $U' = (U^{n_{uu}}/(k_{uu}{}^{n_{uu}} + U^{n_{uu}}))(Z^{n_{zu}}/(k_{zu}{}^{n_{zu}} + Z^{n_{zu}}))(k_{vu}{}^{n_{vu}}/(k_{vu}{}^{n_{vu}} + V^{n_{vu}})) - U$
  71. $V' = (V^{n_{vv}}/(k_{vv}{}^{n_{vv}} + V^{n_{vv}}))(k_{uv}{}^{n_{uv}}/(k_{uv}{}^{n_{uv}} + U^{n_{uv}})) - V$
  Where we used the following parameter values:
  $$k_{ii} = 0.3, n_{ii} = 1, k_{uv} = k_{vu} = 0.3, k_{vx} = k_{xy} = k_{xz} = k_{yz} = k_{zu} = 0.01, n_{uv} = n_{vu} = 3, n_{vx} = n_{xy} = n_{xz} = n_{yz} = n_{zu} = 1$$
- Interaction between a coherent and an incoherent FFL (Fig. 5D):
  72. $X' = (U^{n_{ux}}/(k_{ux}{}^{n_{ux}} + U^{n_{ux}})) - X$
  73. $Y' = (X^{n_{xy}}/(k_{xy}{}^{n_{xy}} + X^{n_{xy}})) - Y$
  74. $Z' = (k_{yz}{}^{n_{yz}}/(k_{yz}{}^{n_{yz}} + Y^{n_{yz}}))(X^{n_{xz}}/(k_{xz}{}^{n_{xz}} + X^{n_{xz}})) - Z$
  75. $W' = (k_{zw}{}^{n_{zw}}/(k_{zw}{}^{n_{zw}} + Z^{n_{zw}})) - W$
  76. $V' = (W^{n_{wv}}/(k_{wv}{}^{n_{wv}} + W^{n_{wv}})) - V$
  77. $U' = (V^{n_{vu}}/(k_{vu}{}^{n_{vu}} + V^{n_{vu}}))(W^{n_{wu}}/(k_{wu}{}^{n_{wu}} + W^{n_{wu}})) - U$
  Where we used the following parameter values:
  $$k_{ux} = k_{xy} = k_{xz} = k_{yz} = k_{zw} = 0.01, k_{vu} = 13.4, k_{wu} = 55.9, k_{wv} = 70.8, n_{ij} = 1$$

Models and parameters used in Figure S4 and S5

As described in the main text and in the Methods section, to model network motifs and hyper-motifs we consider that each node has a linear removal term. Every directed edge from X to Y is considered with the following function that multiplies the production term of Y: $X^{n_{xy}}/(k_{xy}{}^{n_{xy}} + k_{xy}{}^{n_{xy}})$ for a positive interaction and $k_{xy}{}^{n_{xy}}/(k_{xy}{}^{n_{xy}} + k_{xy}{}^{n_{xy}})$ for a negative interaction. We used in Figure S4 the following parameters:

- Figure S4C-F: $k_{ij} = 0.3, n_{ij} = 3$
- Figure S4G-J: $k_{xx} = k_{ij,i \neq j} = 0.3, k_{yy} = k_{zz} = 0.2, n_{ij} = 3$
- Figure S5A-B:
  $$k_{ii} = k_{wi} = k_{iw} = k_{xz} = 0.3, k_{xy} = k_{yz} = 0.01, n_{ii} = n_{wi} = n_{iw} = 3, n_{xy} = n_{xz} = n_{yz} = 1$$
- Figure S5C-D:
  $$k_{ii} = k_{wi} = k_{iw} = 0.3, k_{xy} = k_{xz} = 0.01, k_{yz} = 0.5, n_{ii} = n_{wi} = n_{iw} = 3, n_{xy} = n_{xz} = n_{yz} = 1$$
- Figure S5E-F:
  $$k_{ii} = k_{wi} = k_{xz} = 0.3, k_{iw} = 0.5, k_{xy} = k_{yz} = 0.01, n_{ii} = n_{wi} = n_{iw} = 3, n_{xy} = n_{xz} = n_{yz} = 1$$
- Figure S5G-H:
  $$k_{ii} = k_{wi} = k_{xz} = 0.3, k_{iw} = 0.5, k_{xy} = 0.01, k_{yz} = 0.5, n_{ii} = n_{wi} = n_{iw} = 3, n_{xy} = n_{xz} = n_{yz} = 1$$
